# A new *Caenorhabditis elegans* apurinic/apyrimidinic (AP) endonuclease engaged in rescue from replication stress-induced arrest

**DOI:** 10.1590/1678-4685-GMB-2024-0216

**Published:** 2025-10-31

**Authors:** Seoyun Choi, Hoa Thi Pham, Byungchan Ahn

**Affiliations:** 1 University of Ulsan, Department of Life Sciences, Ulsan, Korea.; 2 The George Washington University, School of Medicine & Health Sciences, Department of Biochemistry & Molecular Medicine, Washington, DC, USA.

**Keywords:** AP endonuclease, 3′-5′ exonuclease, replication stress, cell cycle arrest, Caenorhabditis elegans

## Abstract

Apurinic/apyrimidinic sites are one of the most frequent spontaneous lesions in DNA. Evolutionarily conserved AP endonucleases (ExoIII and EndoIV families) incise the DNA backbone 5′ to the AP site and the cleaved AP sites are subsequently repaired by the base excision repair machinery. AP endonucleases additionally exhibit 3′-5′ exonuclease activity. Novel AP endonucleases that are not member of AP endonuclease families keep being reported and exhibit 3′-5′ exonuclease activity and other important DNA processing. Interestingly, human and mouse WRN helicases contain a 3′-5′ exonuclease domain, but the precise functional roles of the exonuclease activity *in vivo* remain unclear. We searched for WRN-like exonuclease proteins in the *Caenorhabditis elegans* database and found a new gene, *zk1098.3*, which shows a high similarity to human EXD3. Here, we assigned *zk1098.3* to *exd3-1*. We cloned *exd3-1* from an ORF clone and purified the recombinant EXD3-1 protein*.* We found that EXD3-1 displays incision at AP sites and exonucleolytic digestion on the nicked AP site and that EXD3-1 is involved in recovery from replication stress-induced cell cycle arrest. This work suggests that EXD3-1 either plays a role in base excision repair, although the extent of this repair remains to be determined, or has a specialized DNA damage response function.

## Introduction

Apurinic/apyrimidinic (AP or abasic) sites are generated by cleavage of the N-glycosylic bond between the nitrogenous base and the deoxyribose sugar resulting in base loss in DNA strand (Lindahl and Nyberg, 1972; Lindahl, 1993). AP sites are also enzymatically formed in an intermediate step during the repair of damaged DNA by the base excision repair (BER) pathway (Krokan and Bjoras, 2013). The common mutations arising from AP sites are base substitutions, particularly the insertion of adenine opposite the lesion, as well as frameshift mutations and strand breaks. (Loeb and Preston, 1986). BER corrects DNA bases damaged by oxidation, alkylation, and deamination, and is well conserved in many organisms from *Escherichia coli* (*E. coli*) to humans (Krokan and Bjoras, 2013). This repair pathway is initiated by DNA glycosylases which excise damaged or misincorporated bases from DNA strand, resulting in an AP site. Correcting AP sites is then initiated by incision at AP sites by AP endonucleases (Boiteux and Guillet, 2004; Auerbach et al., 2005). There are two families of AP endonucleases in living organisms; based on their homology to the two *E. coli* enzymes exonuclease III (ExoIII) and endonuclease IV (Endo IV) (Mol et al., 2000; Daley et al., 2010). ExoIII was first identified and the ExoIII homologs are present in all kingdoms of life, named apurinic-apyrimidinic endonuclease 2 (APN2) in *Saccharomyces cerevisiae* (Unk et al., 2000) and AP endonuclease 1 (APE1) and APE2 in humans (Demple et al., 1991; Hadi and Wilson III 2000). The *Arabidopsis thaliana* (L.) Heynh genome encodes three ExoIII homologs (Murphy et al., 2009). The ExoIII family enzymes have AP endonuclease, 3′- deoxyribophosphodiesterase, 3′-5′ exonuclease, and 3′-phosphatase activities (Li and Wilson III, 2014). These activities are Mg^2+^-dependent and are exhibited on a wide variety of DNA substrates. ExoIII-deficient *E. coli* mutants were examined. From these, Endo IV was identified. The Endo IV homolog APN1 is present in *Saccharomyces cerevisiae* and *Caenorhabditis elegans* (*C. elegans*) but not in some mammals and *Arabodopsis* (Levin et al., 1991; Hadi and Wilson III, 2000; Carpenter et al., 2007;Murphy *et al.,* 2009). The Endo IV family enzymes exhibit additionally 3′-5′ exonuclease and 3′-dRpase activities, which are Zn^2+^- dependent (Boiteux and Guillet, 2004). These AP endonucleases cleave the DNA phosphodiester bond 5′ to the AP site to produce a 3′-hydroxyl group and a 5′-deoxyribose phosphate. The remaining abasic sugar is removed by a dRpase, leaving a single nucleotide gap. Finally, DNA polymerase adds the correct nucleotide to the DNA strand, and DNA ligase seals the remaining nick in the sugar-phosphate backbone. 

Unlike other multicellular eukaryotes, BER in *C. elegans* has not been well characterized and few studies on BER have been reported (Elsakrmy et al., 2020). Two glycosylases (Shatilla and Ramotar, 2002; Nakamura et al., 2008; Morinaga et al., 2009) and two AP endonucleases have been identified (Shatilla *et al.,* 2005a,b) and characterized biochemically (Shatilla *et al.,* 2005a; Asagoshi et al., 2012; Yang et al., 2012 b ). The identified AP endonucleases are EXO-3 (an ortholog of ExoIII) and APN-1 (an ortholog of Endo IV) (Masson et al., 1996; Shatilla *et al.,* 2005a,b). EXO-3 is an Mg^2+^-dependent enzyme and shows no significant level of 3′-5′ exonuclease activity following incision at AP sites, while APN-1 is a Zn^2+^-dependent enzyme and displays a 3′-5′ exonuclease activity (Yang *et al.,* 2012a) on 3′ ends bearing bulky groups. 

Novel AP endonucleases that do not belong to the ExoIII and Endo IV families keep being reported and also possess 3′-5′ exonuclease activity. However, they contain an additional domain which is involved in other important DNA processing. In *Bacillus subtilus*, DNA polymerase PolX_Bs_ exhibits AP endonuclease activity (Banos et al., 2010). In humans, a novel protein Polynucleotide kinase and aprataxin-like forkhead-associated protein (PALF) that is involved in responding to DNA strand break possesses AP endonuclease activity (Kanno et al., 2007). More recently, TatD (twin-arginine translocation) proteins that help export proteins from the cell also exhibit AP endonuclease activity (Dorival and Eichman, 2023). Those new AP endonucleases also are found to commonly exhibit 3′-5′ exonuclease activity. It is interesting that human and mouse Werner syndrome proteins (WRN) contain both helicase and 3′-5′ exonuclease domains that influences DNA repair events. However, the exact functional roles of the exonuclease *in vivo* remain unclear. Thus, the function of WRN-like 3′-5′ exonuclease remains to be further investigated in other living organisms. To identify orthologs of WRN-like 3′-5′ exonuclease in *C. elegans*, we began by performing a protein BLAST search (NCBI) for homologs of human WRN 3′-5′ exonuclease. This analysis revealed that both human EXD3 (exonuclease 3′-5′ domain containing 3) and EXD2 (exonuclease 3′-5′ domain-containing protein 2) share a high amino acid sequence similarity with the WRN exonuclease domain. Then, we searched for *C. elegans* orthologs of both EXD3 and EXD2 and identified a putative gene *zk1098.3*, which reveals a high level of amino acid sequence similarity to EXD3. We assigned *zk1098.3* as *exd3-1.*

Here, we report that the *exd3-1* gene product, EXD3-1 unexpectedly exhibited Mg^2+^-dependent AP endonuclease and 3′-5′ exonuclease activities. In contrast to typical AP endonucleases, EXD3-1 is involved in response to replication fork-associated DNA damage. 

## Material and Methods

###  Bacterial strains and *C. elegans* strains 

*E. coli* bacterial strains DH5a and BL21AI (Invitrogen, USA) were maintained on Luria-Bertani (LB) agar plates. The wild-type *C. elegans* strain was Bristol N2. A mutant strain (*tm2546*) of *zk1098.3* was generated by the National BioResource Project, (Tokyo, Japan) and carried a 369-bp deletion and a 14-bp insertion covering exons 3 and 4 out of the total 6 exons, resulting in a frame shift. Mutant worms homozygous for this allele are viable (http://www.wormbase.org). Unless otherwise indicated, worms were maintained and propagated at 20 ℃ on nematode growth medium (NGM) agar and fed with streptomycin resistant *E. coli* OP50 bacterial strain. 

### Proteins and antibodies

*E. coli* Endo IV and uracil DNA glycosylase (UDG) enzymes were purchased from New England Biolabs (MA, USA). A mouse monoclonal anti-Glutathione-S-Transferase (GST) antibody was purchased from Ab Frontiers (Korea). The EXD3-1 antibody was prepared by antipeptide antibody production. Two polypeptides derived from Peptide 1 (1^st^~21^st^; MGDTTSSEDVPENKQKSLKFE-c terminus) and Peptide 3 (273^rd^~295^th^; KFFNGQVKQFKFDLEERRDAPKF-c terminus) of EXD3-1 were selected and polyclonal antipeptide antibodies were produced from rabbits (Ab Frontiers, Korea) and further purified by Protein A column. Anti-RPA-1 antibody for *C. elegans* replication protein A-1 (RPA-1) was produced from mouse (Hyun et al., 2012). A horseradish peroxidase (HRP)-conjugated secondary antibody (mouse or rabbit) was purchased from Sigma-Aldrich (USA).

### Construction of a GST-Fusion Protein

*C. elegans zk1098.3* (2355 nucleotides) was referred to this study as *exd3-1*. The gene from ORF clone (Catalog#: OCE1182-202134345, Open Biosystems, UK) was used as a template for PCR amplification of the corresponding gene with two primers (Forward primer: 5′-CACC ATG GGA GAT ACA ACA TCA TCG-3′; Reverse primer: 5′-TTA ACA ATT CAA TTT AAT ATA ATC TTG ATC TGG-3′) and *Pfu* DNA polymerase (Stratagene, USA). PCR product was cloned into the plasmid using pENTR/D/TOPO Cloning kits (Invitrogen, USA). The sequence of *exd3-1*in the resulting plasmid pENTR-exd3-1 was confirmed by DNA sequencing (Figure S1).

The cloned *exd3-1* gene was transferred to the *E. coli* expression vector pDEST15 (Invitrogen, USA) for GST-tagged fusion protein using the Gateway LR Clonase II (Invitrogen, USA) and the resulting plasmid was transformed into *E. coli* BL21AI strain for protein expression.

### Purification of GST-fusion protein

The recombinant GST_EXD3-1 protein was expressed in *E. coli* BL21AI. *E. coli* cells were grown to an OD_600_ of 0.4 in 1 liter of LB broth containing 100 µg/ml ampicillin at 37°C. L-arabinose (Sigma-Aldrich, USA) was added to the culture to a final concentration of 0.2% (w/v) to induce protein expression and were further grown overnight at 22°C. Cultured cells were harvested by centrifugation, suspended in 20 ml lysis buffer (1x PBS, 10% glycerol, 1 mM Phenylmethylsulfonyl fluoride (PMSF), protease inhibitor (1:1000 dilution, Calbiochem, USA) and lysed by sonication (duty cycle 20/output 2, 10 bursts at 10 second intervals, Branson, USA). Lysates were clarified by centrifugation at 10,000×g for 30 minutes at 4°C. Supernatant (after centrifugation) was incubated with Glutathione sepharose 4 fast-flow resins (GE healthcare, USA) with rocking for 3 hours at 4°C. The incubated resin was packed into a column and flow-through was collected using a constant rate (0.25 ml/min). The column was washed with 10 ml of lysis buffer (except protease inhibitors) and with 20 ml of wash buffer 1 (100 mM Tris-HCl at pH 8.0, 150 mM NaCl, 5 mM DTT, 10% glycerol), 0.1% Triton X-100). GST_EXD3-1 was eluted with10 ml of elution buffer (100 mM Tris-HCl at pH 8.0, 150 mM NaCl, 5 mM DTT, 10% glycerol, 0.1% Triton X-100, 1 mM reduced glutathione). A second elution was performed with 5 mM glutathione. Peak fractions containing GST_EXD3-1 were analyzed by SDS polyacrylamide gel electrophoresis (SDS-PAGE) on a 7% separation gel and detected in fraction numbers 5 ~ 8 of first elution buffer. Protein concentrations were determined by a Bio-Rad Protein assay with BSA as a protein standard for the linear range of the assay. GST_EXD3-1was hereafter referred to as EXD3-1.

### Western blot analysis

EXD3-1 (10 ng) was analyzed by SDS polyacrylamide gel electrophoresis (SDS-PAGE) on a 7% separation gel and transferred to Polyvinylidene Fluoride (PVDF) membrane. The membrane was incubated with blocking solution (1% fat-free milk with 1x PBST (1x Phosphate-Buffered Saline (PBS) and 0.1% Tween 20) for 2 hours at 4°C. The membrane was incubated with anti-GST antibody (1:10000 dilution) or anti-EXD3-1 antibody (1:1000 dilution) overnight at 4°C in blocking solution. After washing, the membrane was incubated with a horseradish peroxidase (HRP)-conjugated secondary antibody (1:5000 dilution). Western blot was detected with West Pico kit (Pierce, USA).

### Oligonucleotide substrates

Synthetic 24-mer oligonucleotides with a deoxyribouracil (dU) at position 14 and a complementary strand containing G opposite to U were synthesized by Bioneer (Korea). Synthetic 24-mer oligonucleotides (THF-A) with a Tetrahydrofuran (THF, a synthetic analog of an abasic site) lesion at position 12 and a complementary strand containing A opposite to THF were synthesized by Bioneer (Korea). Oligonucleotides are listed in Table S1 (supplementary information). A single oligonucleotide containing dU or THF was labeled at the 5′-end with [γ-^32^P] ATP (IZOTOP, Hungary). Oligonucleotides were incubated with T4 polynucleotide kinase (New England Biolabs, USA) for 1 hour at 37 ℃, followed by heat-inactivation for 10 minutes at 95 ℃. The labeled oligonucleotides were purified using G-25 Sepharose spin columns (Amersham, USA). Oligonucleotides (10 pmol of labeled oligonucleotides and 30 pmol of complementary oligonucleotides) in PBS were heated at 95°C for 5 min and then cooled down to RT. The resulting duplex oligonucleotides are referred to as dU-G and THF-AP.

### Incision assay

For measuring AP site cleavage activity, DNA substrates were incubated with proteins (EXD3-1 or *E. coli* Endo IV) in 15 µl (final volume) of buffer (40 mM Tris-Cl at pH 8.0, 5 mM DTT, 4 mM MgCl_2_, 0.1 mg/ml BSA, 10% glycerol) at 37°C for indicated times. Reactions for THF-A duplex substrate were stopped with 15 µl formamide loading buffer (95% deionized formamide, 0.025% bromophenol blue, 0.025% xylene cyanole, and 10 mM EDTA). Samples were heated at 95°C for 5 min. Reaction for dU-G duplex substrates was incubated *E. coli* UDG followed by heating at 55°C for 10 min. Then, EXD-3 or *E. coli* Endo IV was incubated at 37°C for the indicted times. Reaction was stopped with formamide loading buffer and heated at 95°C for 5 min before loading onto gels. Reaction products were separated by 8 M urea-17% polyacrylamide gel electrophoresis (PAGE, gel length 16 cm) and viewed by autoradiography. The intensities of DNA bands were measured using NIH ImageJ (available at http://rsb.info.nih.gov/ij/).

### Immunodepletion assay

EXD3-1 protein (1 µg, 8.3 pmol, 30 µl) was incubated with Protein G Dynabeads suspension (50 µl, 1.5 mg, Novex, Thermo Fisher Scientific, USA) followed by application of magnetic field to separate the beads. The resulting supernatant (pre-cleared EXD3-1) was collected. The pre-cleared EXD3-1 (55 µl) was then incubated with anti-GST antibody (10 µg) in immunodepletion buffer (50 mM Tris-HCl at pH 8, 120 mM NaCl, 0.5% NP-40, protease inhibitor; Calbiochem, USA) at 4°C for 1 hour with gentle rotation. Afterward, a fresh Protein G Dynabeads suspension (25 µl) was added to the mixture and incubated for an additional hour at 4°C with gentle agitation. A magnetic field was applied to pull beads to the side of the tube and the supernatant containing immunodepleted protein (65 µl) was carefully collected by pipetting. This supernatant was then assayed for AP site cleavage activity as described above. 

### Embryonic survival after hydroxyurea (HU) and Camptothecin (CPT) treatments

Ten L4-stage animals were grown on NGM agar plates containing indicated concentrations of HU (stock solution; 50 mg/ml in H_2_O, Sigma-Aldrich) at 20°C for 16 hours. Worms were transferred to HU-free NGM agar plates lawn with *E. coli* OP50 and let worms lay eggs at 20°C. After 3-4 hours, the worms were remonved and, the number of total laid eggs was counted. The number of hatched F1 larvae and unhatched eggs in the F1 generation were 24 hours later using a dissection microscope. Embryonic survival was scored as the percentage of hatched F1 larvae dividing by the sum of the numbers of hatched F1 larvae and of unhatched eggs. 

L4-stage animals were grown on NGM plates containing indicated concentrations of CPT (stock 20 mg/ml in 1 N NaOH, Sigma-Aldrich) for 24 hours and then transferred to CPT-free NGM agar plates seeded with *E. coli* OP50, where embryos were laid for 2-3 hours. After 24 hours, the number of total laid eggs was counted. The number of hatched F1 larvae and unhatched eggs in the F1 generation was counted 24 hours later using a dissection microscope. Embryonic survival was scored as the percentage of hatched F1 larvae dividing by the sum of the numbers of hatched F1 larvae and of unhatched eggs. 

### Mitotic germ cell proliferation arrest

To observe nuclear morphology, worms were grown at 20°C to larval stage L4. L4 worms were transferred to a new NGM plate containing HU or CPT and treated for indicated times. Worms were transferred to 1x PBST on a glass plate and dissected worm gonads. Dissected gonads were harvested in a 1.5 ml microcentrifuge tube. Gonads were fixed by incubating with 3% paraformaldehyde for 15 min at room temperature and post-fixed in ice-cold methanol for 5 min at room temperature. 4,6-diamidino-2-phenylindole (DAPI, 1 µg/ml) was added to gonads followed by incubating for 5 min in the dark. Gonads were rinsed three times for 10 min in 1x PBST and then transferred onto 1% agarose-padded slide glass. Gonads were observed under an epifluorescence microscope (Carl Zeiss Axioskop2 plus).

### Immunostaining 

Worms were transferred to 1x PBST buffer supplemented with 0.2 mM levamisol (Sigma-Aldrich, USA) on a glass plate and dissected. Dissected gonads were harvested in a 1.5 ml microcentrifuge tube and fixed in 3% paraformaldehyde for 15 min at room temperature followed by post-fixation with ice-cold methanol at room temperature for 5 min. Gonads were treated in 1x PBSX (1x PBS and 0.1% Triton-X) for permeabilization followed by washing with 1x PBST. Blocking was performed by incubating the samples in 1x PBSTB (1x PBST and 0.5% BSA) for 30 min. Primary antibody (anti-RPA-1 antibody, a 1:1000 dilution in 1x PBSTB) was added and incubated overnight at 4°C. Samples were washed three times for 10 min in 1x PBST. Binding of secondary antibodies was performed for 2 hours at room temperature with Alexa Fluor-conjugated goat anti-mouse secondary antibodies (used in a 1:2000 dilution, Molecular probes) and washed three times for 10 min in 1x PBST. After staining with DAPI (1 µg/ml), Gonads were mounted on 1% agarose pads and observed under an epifluorescence microscope (Carl Zeiss Axioskop2 plus).

### Multiple sequence alignment

The human WRN (NP_000544.2), EXD3 (NP_060290), and EXD2 (NP_001180289) protein sequence information were obtained from NCBI (https://www.ncbi.nlm.nih.gov/). *C. elegans mut-7* (UniProtP34607) and *zk1098.3* (UniProtP34603) protein information was obtained from Uniprot (https://www.uniprot.org/).

Multiple sequence alignment was performed with a protein BLAST search (NCBI) and Expresso (a multiple sequence alignment server), an alignment method through structural information and T-Coffee multiple sequence alignment software (http://tcoffee.crg.cat/) available online.

## Results

### Purification of the EXD3-1 protein that contains a 3'-5' exonuclease domain similar to the human EXD3

The sequence of human WRN exonuclease domain (amino acids 1 to 333) was used to perform a protein BLAST search (NCBI). This search identified two homologs of WRN exonuclease domain: human EXD3 with an expect value (e = 2×10^-11^) and EXD2 with an expect value (e = 3×10^-13^). Multiple sequence alignment using Expresso showed that the WRN exo domain (amino acids 57-235) was aligned with corresponding exo domains in EXD3 (amino acids 373-568) and EXD2 (amino acids 82-258) (Figure S2A). These three sequences were then used to search for *C. elegans* orthologs using a protein BLAST search, which resulted in two putative orthologs: *mut-7* (UniProtP34607) and *zk1098.3* (UniProt P34603; an expect value (e = 8×10^-8^). Subsequent sequence alignment with Expresso revealed that amino acid residues 422~ 619 of *zk1098.3* are high similarity to the exonuclease domain of human EXD3 (Figure S2B). 

**Figure 1-:**
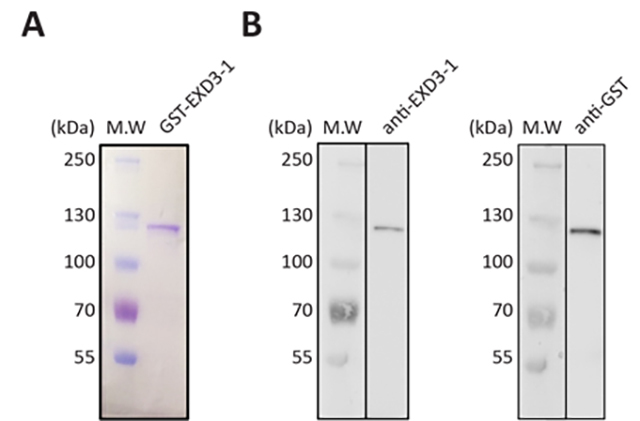
Recombinant GST_EXD3-1 protein. (A) SDS-PAGE analysis of purified EXD3-1 on a 7.5% separating gel. M (kDa), relative molecular mass markers; EXD3-1, GST_EXD3-1 protein (1 µg). Image of SDS-PAGE was analyzed using ImageJ (NIH) and the purity of the purified EXD3-1 was determined to be ~95%. (B) Western blotting of EXD3-1. EXD3-1 was separated by SDS-PAGE and subjected to western blot analysis using anti-EXD3-1 or anti-GST antibodies followed by chemilluminescence. A single protein band detected by western blot corresponds to the migration of the single protein band in (A).

The ZK1098.3 protein consists of 784 amino acids and has a calculated molecular weight of 91,135. In this study, *zk1098.3* was designated *exd3-1*. The full length *exd3-1* gene from *C. elegans* was amplified by PCR using *C. elegans* ORF clone as a template. The PCR product was then cloned into pDEST15 to express GST-fusion EXD3-1 (GST_EXD3-1, hereafter referred to as EXD3-1) in *E. coli*. The sequence of *exd3-1* was confirmed by DNA sequencing (Figure S1). The recombinant protein was purified to approximately 95% homogeneity (Figure 1A). SDS-PAGE analysis on a 7% polyacrylamide gel revealed a single protein band at ∼120 kDa, consistent with the expected molecular weight of the fusion protein (26.9 kDa GST + 91.1 kDa EXD3-1). Western blot analysis using polyclonal anti-EXD3-1 and monoclonal anti-GST antibodies confirmed the presence of an immunoreactive protein at approximately 120 kDa (Figure 1B), in agreement with the SDS-PAGE results. It is noted that GST is commonly used for affinity chromatography and protein purification (Harper and Speicher, 2011) and is primarly involved in the detoxification process. To date, GST has not been reported to exhibit nuclease activity.

### EXD3-1 introduces nicks at THF AP sites

**Figure 2 -:**
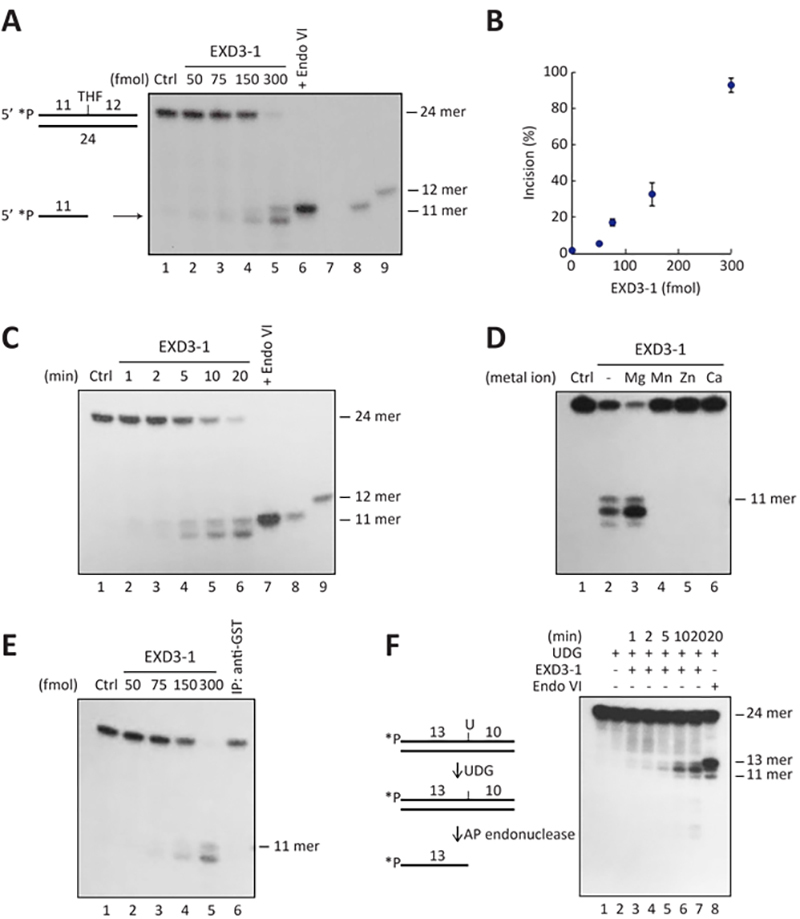
EXD3-1 exhibited Mg-dependent incision on duplex DNA containing AP site. (A) Incision activity of EXD3-1 on a THF-AP containing substrate. Duplex DNA (24-mer) containing a THF-AP site and product lengths are displayed on the left side of autoradiograph. Incision reaction (15 μl) that contains ^32^P-labeled AP substrates (15 fmol) and the indicated amounts of EXD3-1 was performed at 37°C for 20 min. Reaction products were separated by 8 M urea-17% PAGE and DNA fragments were visualized by autoradiography. Lane 1, Ctrl, DNA only and no protein; lanes 2-6, the indicated amounts of EXD3-1; lane 7, *E. coli* Endo IV (0.1 U); lane 8, an 11-mer size marker; lane 9, a 12-mer size marker. (B) Quantitation of incision at THF-AP site. Three independent experiments from (A) were conducted and the autoradiographic band intensities were quantified with NIH ImageJ software. Incision percentage was calculated and normalized to DNA only (Ctrl). 33% incision was detected at 150 fmol and 93% at 300 fmol.(C) Time-course incision by EXD3-1. A ^32^P-labeled THF-AP substrate (15 fmol) were incubated with EXD3-1 (300 fmol) for the indicated times. Lane 1, Ctrl, DNA only and no protein; lanes 2-6, the indicated times (min); lane 7, *E. coli* Endo IV (0.1 U); lane 8, an 11-mer size marker; lane 9, a 12-mer size marker. (D) Effects of divalent metal ions on incision activity of EXD3-1. A ^32^P-labeled THF-AP substrate (15 fmol) was incubated with EXD3-1 in the presence of each of the indicated metal ions (4 mM). Reaction products were separated by 8 M urea-17% PAGE and DNA fragments were visualized by autoradiography. The length of the fragment is indicated on the right side of the gel. Lane 1, Ctrl, DNA only and no protein; lane 2, no metal ion; lane 3, in the presence of MgCl_2_; lane 4, MnCl_2_; lane 5, ZnCl_2_; lane 6, CaCl_2_. (E) Loss of AP incision activity in immunodepleted EXD3-1. Pre-cleared EXD3-1 protein (prepared as described in Methods) was incubated with anti-GST antibody, followed by incubation with Protein G beads. After applying a magnetic field, supernatant was collected and assayed for cleavage activity. Lane 1, no protein; lanes 2-5, indicated amounts of EXD3-1; lane 6, immunodepleted supernatant (2 µl, equivalent to 300 fmol of EXD3-1). Fragment lengths are indicated on the right side of the autoradiograph. (F) EXD3-1 exhibited incision activity on a natural abasic site. Duplex DNA (24-mer) containing a dU-G pair (at 14-nt from the 5′-end) was treated with*E. coli*UDG (2x10^-5^ U) followed by heating 55°C, leaving a natural AP site (lane 2). The resulting AP-containing DNA (15 fmol) was incubated with EXD3-1 (lanes 3-7) or *E. coli* Endo IV (lane 8). Reaction products were separated by 8 urea-17% PAGE and the DNA fragments were visualized by autoradiography. Lane 1, UDG-untreated DNA; lane 2, UDG-treated DNA; lanes 3-7, incubation with EXD3-1 (300 fmol) for the indicated times; lane 8, *E. coli* Endo IV (0.1 U), producing a 13-mer product by its cleavage. Fragment lengths are indicated on the right side of the autoradiograph.

Some proteins containing 3′-5′ exonuclease activity are multifunctional enzymes that also show additional functions, such as AP endonuclease activity. To investigate whether EXD3-1 possesses AP endonuclease activity, we used THF-AP duplex, which contained a 5′-[^32^P]-labeled oligonucleotide with THF (abasic analog) at position 12 (Figure 2A, Table S1, and Figure S3). To confirm that THF acts as an AP lesion, we first incubated the substrate with *E. coli* Endo IV, which cleaves the phosphodiester bond 5′ next to a THF site, leaving a single-stranded DNA break (Hosfield et al., 1999). As expected, an 11-mer cleavage product was produced (Figure 2A, lane 7). Surprisingly, EXD3-1 also efficiently introduced nicks at the THF site, generating the same 11-mer cleavage product (Figure 2A, lanes 2-6). In addition, a shorter fragment was observed, which is likely resulted from the exonuclease activity of EXD3-1 acting on the nicked THF site. EXD3-1 showed a clear preference for the THF-containing double-stranded DNA (dsDNA), as no incision was detected in the control duplex DNA lacking THF lesion (Figure S3, blunt-end).

The incision of THF-AP substrate was observed in a protein concentration-dependent manner (Figure 2A, lanes 2-6) and the incision linearly increased with increasing concentrations (Figure 2B). The time course assay of incision revealed that the incised product was formed linearly over the time points (Figure 2C, lanes 2-6). 

Members of the ExoIII family AP endonucleases, from *E. coli* to humans, are divalent metal ion-dependent enzymes that require Mg^2+^ (Kow, 1989; Shatilla et al., 2005 a ; Tsutakawa et al., 2013), whereas Endo IV members utilize Zn^2+^ (Levin et al., 1991; Hosfield et al., 1999). In our study, the incision reactions shown in Figures 2A-2C were initially performed with MgCl_2_, following conditions previously reported for other AP endonucleases (Shatilla *et al.,* 2005a; Gelin et al., 2010; Lee et al., 2022). Given that other AP endonucleases also exhibit activity in the presence of Mn^2+^ (Schermerhorn and Delaney, 2013; Abeldenov et al., 2015), we assessed the effect of various divalent metal ions on EXD3-1-mediated incision of THF-AP substrate and its exonucleolytic degradation (Figure 2D). In the absence of any divalent metal ions, EXD3-1 exhibited weak cleavage activity on THF-AP substrates (Figure 2D, lane 2). However, this incision was abolished in the presence of the metal-chelating agent EDTA (Figure S4, lane 2), indicating that diavalent metal ions are in the purified EXD3-1 sample. When 4 mM MgCl_2_ was added to the incision reaction in the absence of EDTA, the incision and exonucleolytic degradation were significantly enhanced (Figure 2D, lane 3) compared to the reaction without added ions (Figure 2D, lane 2). The addition of both EDTA and MgCl_2_ eliminated the activity, further confirming the requirement of Mg^2+^ for EXD3-1 enzyme function (Figure S3, lane 3). In contrast, no cleavage was observed when other divalent metal ions (CaCl_2_, MnCl_2_, or ZnCl_2_) were added (Figure 2D, lanes 4-6). These results indicate that EXD3-1 is metalloenzyme whose incision and exonucleolytic digestion activities are specifically dependent on Mg^2+^. 

To further confirm that the incision activity observed is due to EXD3-1 and not from bacterial contamination, EXD3-1 protein was immunodepleted from the solution using anti-GST antibody. The resulting supernatant was assayed for AP site incision. No incision was detected (Figure 2E, lane 6), indicating that the detected products were indeed generated by EXD3-1. If bacterial contamination were responsible for the activity, it would not be removed by the anti-GST antibody, and incision of the THF-AP substrate would still have been observed in EXD3-1. The absence of activity in immunodepleted supernatant strongly supports that the enzymatic activity is due to EXD3-1. 

### EXD3-1 incises natural AP sites 

We next tested whether EXD3-1 can incise DNA strand containing a natural AP site. To do this, we constructed a 24-mer duplex substrate that contains dU-G base pair (Figure 2F, Table S1). A natural AP site was generated by treating the substrate with *E. coli* UDG, which excises uracil from dsDNA (Lindahl, 1974)*.* The resulting AP site was then cleaved by Endo IV, which cuts the phosphodiester bonds at AP sites, generating a 3′-hydroxyl group and a 5′-terminal sugar phosphate. As expected, a 13-mer product was detected (Figure 2F, lane 8). 

When EXD3-1 was incubated with the UDG-treated substrates over various time intervals, single-strand break was detected on the AP-containing strand (Figure 2F, lanes 3-7), indicating cleavage of the phosphodiester bond 5′ to AP site. In addition, DNA fragments of 12 and 11 nucleotides, shorter than the 13-mer, were detected. These shorter products likely result from exonucleolytic degradation of the strand after the initial incision at AP site (Figure 2F, lanes 3-7), similar to pattern observed with THF-AP substrate (Figure 2). As a control, EXD3-1 did not incise the UDG-untreated substrate, was a blunt-ended, normal oligonucleotide duplex (data not shown). These results suggest that EXD3-1 possesses AP endonuclease activity and can incise natural AP sites following the action of DNA glycosylases.

### *exd3-1*(*tm2546*) mutants are sensitive to HU and CPT 

*C. elegans* (*tm2546*), generated by the National BioResource Project, (Tokyo, Japan), is likely to be null due to a frame shit. Protein expression in this mutant was analyzed by western blotting (Figure S5). No EXD3-1 protein was detected. However, this mutant developed as normal as N2 under normal growth condition (Figure S6).

**Figure 3 -:**
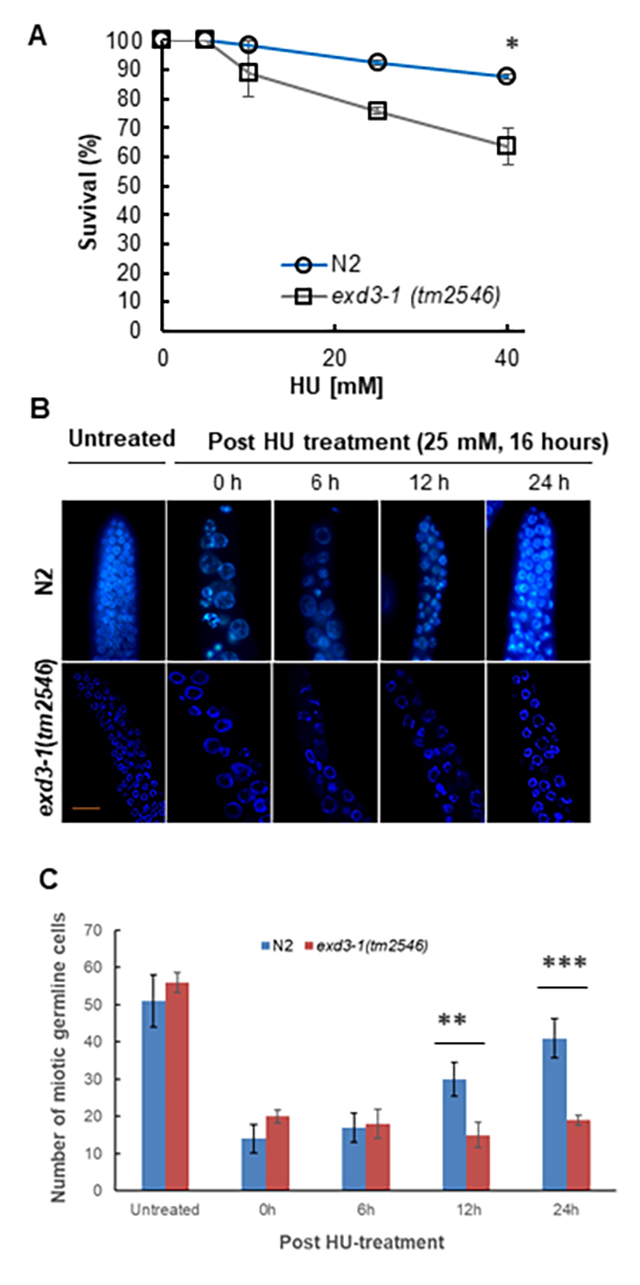
*exd3-1* (*tm2546*) worms were defective in recovery from cell cycle arrest induced by HU. (A) N2 and *exd3-1*(*tm2546*) worms were grown on NGM plates containing the indicated concentrations of HU from the L4 stage for 16 hours and they were placed to HU-free plates to lay eggs for 3 hours. Total laid eggs (200-300) were counted. After 24 hours, the number of hatched F1 larvae and unhatched eggs in the F1 generation were counted. Embryonic survival were scored as the percentage of hatched F1 larvae. Graphs represent mean ± SD from three independent experiments. * indicates *p*-value < 0.003 by Student’s *t*-test. (B) Representative images of germ cell nuclei of indicated worms stained with DAPI. L4-staged worms were transferred to NGM plates following a 16-hour exposure to HU and allowed recovery for indicated times (post HU-treatment). Gonads were then dissected from worms and stained with DAPI. No; no drug treatment. Scale bars, 10 µm. (C) Mitotic germ cell nuclei within 50 μm of a gonad tip were counted from 10 germlines of indicated worms (HU-treatment). Graphs represent mean ± standard deviation from three independent experiments. ** indicates *p*-value < 0.003 and *** indicates *p*-value < 0.0001 by Student’s *t*-test. Blue, N2; red, *exd3-1*(*tm2546*).

Having established the enzymatic activities of EXD3-1, we next examined its response to DNA damage. We measured the survival of embryos to quantify sensitivity of *exd3-1* mutants to DNA damaging agents. HU (DNA replication inhibitor) treatment began at the late L4 larval stage for 16 hours at 20 ℃. The worms were then placed on HU-free NGM plates to lay eggs (200 - 300 eggs) for 2 to 3 hours. The embryonic survival percentage was calculated by dividing the number of hatched eggs by the total number of laid eggs. At 25 mM HU, N2 exhibited 92% survival in our experimental conditions, whereas *exd3-1*(*tm2546*) worms exhibited 75% survival (*P* < 0.003) (Figure 3A), indicating that *exd3-1* (*tm2546*) mutants were more sensitive to HU than N2. 

**Figure 4 -:**
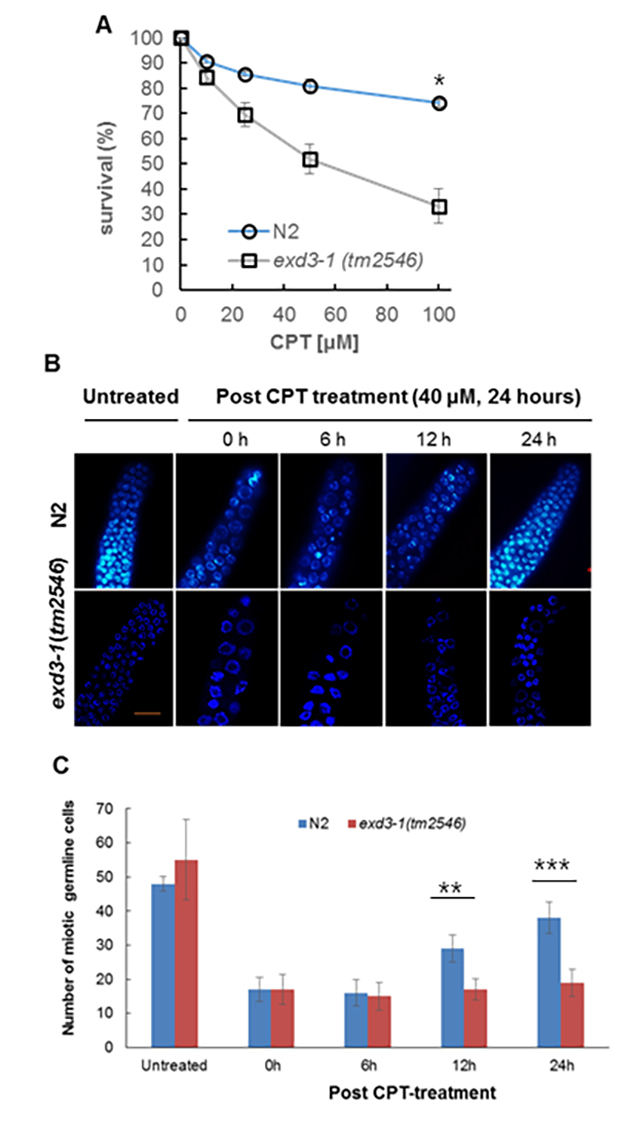
*exd3-1* (*tm2546*) worms were defective in recovery from cell cycle arrest induced by CPT. (A) N2 and *exd3-1*(*tm2546*) worms were grown on NGM plates containing the indicated concentrations of CPT from the L4 stage for 24 hours and they were transferred to CPT-free plates to lay eggs (200 - 300) for 3 hours. After 24 hours, embryonic survival was scored as the percentage of hatched F1 larvae as in (A). Graphs represent mean ± SD from three independent experiments. * indicates *p*-value < 0.004. (B) Representative images of germ cell nuclei of indicated worms stained with DAPI. L4-staged worms were transferred to NGM plates following a 24-hour exposure to CPT and allowed recovery for indicated times (post CPT-treatment). Gonads were then dissected from worms and stained with DAPI. No; no drug treatment. Scale bars, 10 µm. (C) Mitotic germ cell nuclei within 50 μm of a gonad tip were counted from 10 germlines of indicated worms (CPT-treatment). Graphs represent mean ± standard deviation from three independent experiments. ** indicates *p*-value < 0.0001 and *** indicates *p*-value < 0.0001 by Student’s *t*-test. Blue, N2; red, *exd3-1(tm2546)*.

CPT (a topoisomerase inhibitor) treatment began at the late L4 larval stage for 24 hours at 20 ℃. Then, the embryonic survival percentage was calculated. At 40 μM CPT, N2 exhibited 80% embryonic survival of N2, whereas *exd3-1*(*tm2546*) worms exhibited 50% survival (*P* < 0.004) (Figure 4A), indicating that *exd3-1*(*tm2546*) mutants were more sensitive to CPT than N2. 

### Recovery from a replication stress-induced cell cycle arrest 

The *C. elegans* hermaphrodite germ line is a useful model for studying DNA damage response. Exposure of worms to DNA damaging agents, such as HU, CPT, or ionizing radiation causes a transient halt in cell cycle progression in the mitotically proliferating zone (Gartner et al., 2000). The cell cycle arrest leads to a decreased number of mitotic germ cells and enlarged mitotic germ cells since cellular and nuclear growth continues to occur (Craig et al., 2012). HU depletes the nucleotide pool, which inhibits the DNA polymerase and leads to formation of ssDNA. A short HU treatment results in stalled replication forks that retain the ability to restart for some time before being inactivated, whereas a prolonged HU treatment leads to fork collapse, a process that creates a broken DNA end (Petermann et al., 2010; Mason et al., 2019). Worms were exposed for a long HU block (16-hours HU treatment), then we analyzed cell cycle arrest and recovery from the arrest after HU treatment. The morphologies of DAPI-stained mitotic germline nuclei were observed (Figure 3B). Wild-type N2 worms stopped dividing as revealed by enlarged mitotic nuclei and an overall reduction in the number of nuclei in the mitotic compartment of the germline (Figure 3B; N2-0 h post HU-treatment, 3C-0 h; 14 ± 3.8, nuclei number mean ± S.D). Cell cycle arrest was also evident in *exd3-1(tm2546)* mutants (Figure 3B; *tm2546-*0 h post HU-treatment, 3C-0 h; 20 ± 1.7, nuclei number mean ± S.D). Worms were transferred to HU-free NGM plates to release cell cycle arrest. In wild-type N2 germ cells, HU-induced cell cycle arrest appeared to be reversible since the volume of the nuclei gradually decreased over times and the number gradually increased by 24 hours after removal from HU (Figure 3B, post HU-treatment, 3C-24 h; 41 ± 5.2, nuclei number mean ± S.D). In contrast, germ cells in *exd3-1*(*tm2546*) mutants failed to reverse to those in HU-free worms since the shape of nuclei at 24 h post HU-treatment was shown to be abnormal (Figure 3B, post HU-treatment) and the decreased number remained unchanged (Figure 3C-24 h; 19 ± 1.4, nuclei number mean ± S.D, *P < 0.0001*). This result indicates that EXD3-1 is somehow involved in the recovery from collapsed replication forks, which are induced by long HU blocks. 

To induce collapsed replication, worms were treated with CPT (Hyun et al., 2016). Wild-type N2 and *exd3-1*(*tm2546*) worms showed enlarged germ nuclei and decreased number of nuclei (Figure 4B-0 h post CPT- treatment, 4C-0 h; 16 ± 3.9, nuclei number mean ± S.D for N2, 17 ± 4.4, nuclei number mean ± S.D for *exd3-1*(*tm2546*)), indicating that cell cycle arrest was induced. To investigate recovery from CPT-induced cell cycle arrest, worms were transferred to CPT-free plates. N2 showed increased number of mitotic nuclei, similar to that in CPT-untreated worms (Figure 4B-24 h post CPT-treatment, 4C-24 h; 38 ± 4.6, nuclei number mean ± S.D). However, the recovery was delayed in *exd3-1*(*tm2546*) worms compared to the size and number in N2 worms (Figure 4B-24 h post CPT-treatment, 4C-24 h; 19 ± 3.9, nuclei number mean ± S.D*, P < 0.0001*), indicating that that EXD3-1 is somehow involved in the recovery from collapsed replication forks induced by CPT treatment.

**Figure 5.**
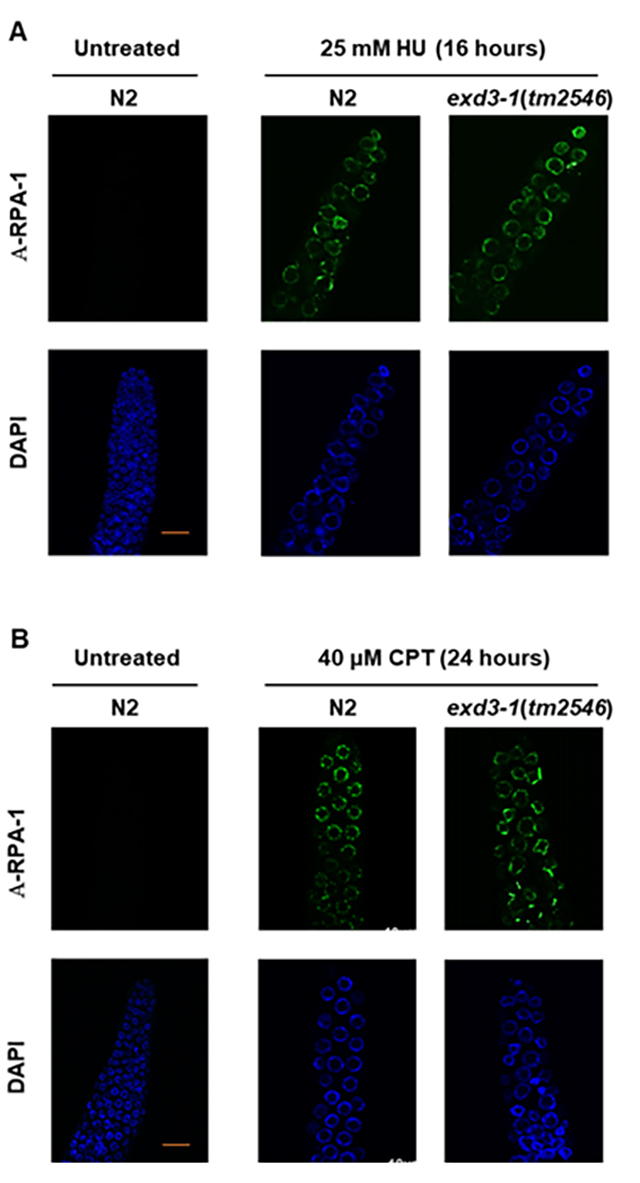
RPA-1 foci were formed normally in *exd3-1*(*tm2546*) worms following exposure to HU and CPT. (A) Gonads of L4 stage worms were separated and immunostained for RPA-1 (green foci) 16 hours after HU (25 mM) treatment and then stained with DAPI. Mitotic region of *C. elegan* gonads was observed by fluorescence microscopy. Scale bars, 10 µm. (B) Gonads of L4 stage worms were separated and immunostained for RPA-1 24 hours after CPT (40 µM) treatment and then stained with DAPI. Mitotic region of *C. elegans* gonad was observed by fluorescence microscopy. Scale bars, 10 µm.

When single-stranded DNA (ssDNA) gaps at stalled forks and ssDNA overhangs at collapsed forks, RPA proteins bind to these ssDNA regions, forming foci, subsequently recruiting DNA damage checkpoint activation proteins (Garcia-Muse and Boulton, 2005). These proteins are involved in the DNA damage responses and repair pathways (Gartner and Engebrecht, 2022). To assess this, we examined RPA-1 foci formation in germline mitotic nuclei after 16 hours HU-treatment or 20 hours CPT-treatment. RPA-1 foci (green) were detected in N2, indicating that both treatments generate ssDNA regions (Figures 5A and B). Similar RPA-1 foci were observed in *exd3-1*(*tm2546*) (Figures 5A and B). These results suggest that RPA-1 responds to both stalled and collapsed replication forks independently of EXD3-1. However, since recovery from cell cycle arrest induced by fork collapse was impaired in *exd3-1*(*tm2546*) worms (Figures 4A and B), this suggests that EXD3-1 is involved in the rescue of collapsed replication forks, likely by promoting the formation of DNA structures competent for the recovery process.

## Discussion

This study shows that a novel *C. elegans* EXD3-1 protein incises the phosphodiester bond 5′ to abasic analog THF sites and natural AP sites. Additionally, EXD3-1 exhibits 3′-5′ exonuclease activity, excising nucleotides from the 3′ end following incision at AP sites. Of the two known *C. elegans* AP endonuclease, APN-1, has been reported to possess 3′-5′ exonuclease that targets 3′ ends bearing bulky groups, but not the 3′ end of the strand generated after AP site incision (Yang et al., 2012b). The other AP endonuclease, EXO-3, has shown no significant 3′-5′ exonuclease activity following incision at AP sites (Shatilla et al., 2005a). Thus, our work suggests that EXD3-1 differs functionally from the two *C. elegans* AP endonucleases. Moreover, the amino acid sequence of EXD3-1 shows no significant similarity to either of the two *C. elegans* AP endonucleases.

Shorter fragments than the 11-mer product could be generated through two scenarios. First, EXD3-1 may introduce multiple endonucleolytic incision 5′ to a THF site, producing shorter fragments-suggesting that EXD3-1 possesses solely as AP endonuclease. Second, EXD3-1 could excise nucleotides exonucleolytically from the remaining strand following incision at the THF site, indicating that EXD3-1 possesses both AP endonuclease and 3′-5′ exonuclease activities. Previous crystal structures and mutational analyses of AP endonucleases have provided mechanistic insights indicating that their endonuclease and exonuclease activities can be regulated within a single active site (Whitaker et al., 2018; He et al., 2020; Liu et al., 2021; Lee et al., 2022). This shared active site in one domain likely facilitates coordinated processing, enabling continuous digestion following AP site incision. Supporting this model, the coupling of the two activities has been observed in ExoIII (Kaneda et al., 2006; Lee *et al.,* 2022), which produces exonucleolytic products. However, human APE1, *H. pylori* AP endonuclease, and *Bacillus subtilis* ExoA have shown that the incision efficiency is the major activity of AP endonucleases (Shida et al., 1999; Gelin et al., 2010; Turgimbayeva et al., 2018).

The10-mer fragment detected in Figure 2A likely represents a product of this coupled activity by EXD3-1. Moreover, smaller fragments (10-mer, 9-mer, and 8-mer) than the 11-mer were observed when higher concentrations of THF-AP substrates were used (Figure S3, THF-AP). Notably, exonucleolytic degradation of a 3′-recessed ends was also observed in 5′-overhang dsDNAs (Figure S3, 5′OH). Taken together, these findings suggest that EXD3-1 digests nucleotides from the strand remaining after AP incision. However, we cannot fully rule out the possibility of multiple endonuleolytic cuts. Further investigation is needed to identify the excised 3′- terminal nucleotides. 

This study also revealed that EXD3-1 requires Mg^2+^ for both incisions at AP sites and exonucleolytic degradation of the resulting strand. Other divalent metal ions were unable to substitute for Mg^2+^ in incisions at AP sites. This dependence of Mg^2+^ is reminiscent of ExoIII family AP endonucleases, which similarly utilize Mg^2+^ for both their endonuclease and exonuclease activities (Shatilla et al., 2005 a ; Lipton et al., 2008; Freudenthal et al., 2015; Turgimbayeva et al., 2018; He et al., 2020). When we tested the AP incision activity of EXD3-1 across a range of MgCl_2_ concentrations (1-8 mM), a constant level of incision was observed (data not shown). Based on this, we used 4 mM MgCl_2_ for incision reactions in this study, aligning with typical conditions for ExoIII family AP endonuclease, which function optimally at 4-5 mM MgCl_2_ (Kow 1989; Shatilla *et al.,* 2005a; Tsutakawa et al., 2013). Moreover, their activity remained stable within a range of 2-8 mM MgCl_2_. 

Interestingly, novel AP endonucleases distinct form the class II AP endonuclease family have been identified in bacteria and humans, and these enzymes possess 3′-5′ exonuclease activity. Human TatD proteins (TATDN1 and TATDN3) and *E. coli* TatD proteins (TatD, YjjV and YcfH) have been shown to possess both AP endonuclease and exonuclease activities (Dorival and Eichman 2023). In *Bacilius subtilus,* DNA polymerase X (PolX_Bs_) exhibits an AP endonuclease activity, which is genetically linked to exonuclease function (Banos et al., 2010). Additionally, human PALF has shown to possess both endo- and exonuclease activities against abasic sites (Kanno et al., 2007). In this study, we report that EXD3-1, which is distinct from the class II AP endonuclease, possesses both AP endonuclease and 3′-5′ exonuclease activities (Figures 2 and 3). 

This study revealed that *exd3-1(tm2546)* mutants failed to resume cell cycle progression following cell cycle arrest induced by collapsed forks, although they exhibited a normal cell cycle arrest induced by HU. Prolonged HU-treatment (16 to 24 hours) leads to fork collapse, which must be rescued for recovery (Saintigny et al., 2001). Cleavage of a stalled fork results in fork collapse and subsequently generates a double-strand break (DSB) (Hanada et al., 2007; Mayle et al., 2015). At the DSB, replication does not restart directly. Instead, the break is resected to generate an ssDNA overhang, which is then repaired via homologous recombination, such as double Holiday junction formation, synthesis-dependent strand annealing, or through nonhomologous end joining (Petermann et al., 2010;Alexander and Orr-Weaver 2016). Therefore, the lack of recovery in *exd3-1* mutants after prolonged HU treatment suggests that induced DSBs may not be properly resected, or that key components required to terminate checkpoint-mediated arrest after repair are missing or dysfunctional. 

When worms were exposed for a short HU block (6-hour HU-treatment), wild-type N2 worms showed a transient arrest in nuclei division, as indicated by enlarged mitotic nuclei and a reduced number of nuclei in the mitotic compartment of the germline (Figure S7). A similar cell cycle arrest was also observed in *exd3-1*(*tm2546*) mutants (Figure S7), suggesting that *exd3-1*(*tm2546*) mutants have an intact S-phase checkpoint capable of inducing cell cycle arrest. To assess recovery, worms were transferred to HU-free NGM plates to release the arrest. In both N2 and in *exd3-1*(*tm2546*) germ cells, the HU-induced cell cycle arrest appeared to be reversible: nuclear volume gradually decreased, and the number of nuclei increased over time, with significant recovery observed by 18 hours after HU removal. Since short HU-treatment can generate ssDNA regions at the stalled replication forks (Petermann et al., 2010), these results indicate that EXD3-1 is not required for recovery from stalled forks.

The involvement of EXD3-1 in the cellular response to DSBs is further supported by its foci formation after prolonged HU-treatment. We analyzed EXD3-1 foci using anti-EXD3-1 antibody after 16 hours of HU treatment (Figure S8). EXD3-1 foci were observed in N2 germline nuclei, while no foci were detected in *exd3-1(tm2546)* mutants, consistent with the absence of EXD3-1 protein expression as confirmed by western blot analysis (Figure S4). Since DSBs are known to be induced at blocked replication forks, the formation of EXD3-1 foci suggests that EXD3-1 may be one of proteins involved in responding or processing collapsed forks. Interestingly, human PALF, which possesses AP endonuclease activity, has also reported to play a role in DNA strand break response (Kanno et al., 2007). Its AP endonuclease activity generates single-strand nicks, which can recruit other DNA damage response factors, such as poly(ADP-ribose) polymerase 1 (PARP1) (Abbotts and Wilson III, 2017). Human PALF protein has also shown to interact with and activates PARP1 (Kanno *et al.,* 2007). Moreover, AP endonuclease and 3′-5′ exonuclease activities of TATDN do not appear to function in canonical BER, but rather play more specialized roles in DNA repair by interacting with other repair factors. Supporting this idea, both AP endonuclease and 3′-5′ exonuclease activities could contribute to the cell cycle and developmental phenotypes observed in zebrafish TATDN1 knockouts (Yang et al., 2012a). Together, our work raises the possibility that EXD3-1 may participate in response to collapsed replication forks through interacting with other repair factors. However, its precise function remains to be elucidated. 

Here, we report a novel protein that has AP endonuclease activity and additionally responds to replication fork-associated DNA damage.

## Supplementary material

The following online material is available for this article:

Table S1 **-**
Oligonucleotide sequences.

Figure S1 **-**
Sequencing of the *exd3-1* gene

Figure S2 **-**
Multiple sequence alignment of 3′-5′ exonuclease domains in various proteins.

Figure S3 **-**
Nuclease activities of EXD3-1 on various duplex DNA substrates.

Figure S4 **-**
Effects of EDTA (metal ion chelator) and divalent metal ions on incision of AP site by EXD3-1.

Figure S5 **-**
Detecting EXD3-1 expression in worms by western blot analysis.

Figure S6 **-**
Larval development of N2 and *exd3-1*(*tm2546*) worms.

Figure S7 **-**
RPA-1 foci were formed normally in *exd3-1*(*tm2546*) worms following 6-hour exposure to HU.

Figure S8 **-**
Formation of EXD3-1 foci after HU treatment.

## Data availability

 The datasets generated and/or analyzed during the current study are available from the corresponding author upon reasonable request.
