# Supplementary material for: A new Caenorhabditis elegans apurinic/apyrimidinic (AP) endonuclease engaged in rescue from replication stress-induced arrest
Source: Genet Mol Biol. 2025 Oct 31;48(3):e20240216. doi: 10.1590/1678-4685-GMB-2024-0216 (PMC12582537; doi:10.1590/1678-4685-GMB-2024-0216)
Supplement: Table S1 - [file 1415-4757-GMB-48-3-e20240216-s1.pdf]

Genetics and Molecular Biology

https://doi.org/10.1590/1678-4685-GMB-2024-0216

Supplementary Material to: A new *Caenorhabditis elegans* purinic/apyrimidinic (AP) endonuclease engaged in rescue from replication stress-induced arrest

Table S1 - Oligonucleotide sequences (5'→3').

1. 5′OH (24-mer)	CGC TAG CAA TTT ACT GTC CAA CTG
2. 5′OH_comp (36-mer)	TTT TTT TTT TTT CAG TTG GAC AGT AAA TTG CTA GCG
3. THF-AP (24-mer)	CGC TAG CAA TT THF ACT GTC CAA CTG
4. dU (24-mer)	CGC TAG CAA TTT AdUT GTC CAA CTG
5. Comp (24-mer)	CAG TTG GAC AGT AAA TTG CTA GCG
