# Supplementary material for: A new Caenorhabditis elegans apurinic/apyrimidinic (AP) endonuclease engaged in rescue from replication stress-induced arrest
Source: Genet Mol Biol. 2025 Oct 31;48(3):e20240216. doi: 10.1590/1678-4685-GMB-2024-0216 (PMC12582537; doi:10.1590/1678-4685-GMB-2024-0216)
Supplement: Figure S1 - [file 1415-4757-GMB-48-3-e20240216-s2.pdf]

Genetics and Molecular Biology

https://doi.org/10.1590/1678-4685-GMB-2024-0216

Supplementary Material to: A new *Caenorhabditis elegans* purinic/apyrimidinic (AP) endonuclease engaged in rescue from replication stress-induced arrest

Figure S1 - Sequencing of the *exd3-1* gene.

The ex*d3-1* gene was sequenced using the Capillary Electrophoresis Sequencing (CES) automation system (Macrogen, Korea). Initial sequencing of the plasmid (pENTR-*exd3-1*) was performed with M13 forward (GTAAAACGACGGCCAGT) and T7 promoter (TAATACGACTCACTATAGGG) primers, followed by internal sequencing using a gene- specific internal primer (GAAACTGATGAGACGGTCAATG).

The resulting sequence was identical to the *zk1098.3* reference sequence in WormBbase, except for a silent mutation at the 62th serine codon (TCA to TCT: shown in blue). Moreover, the sequencing data included the primer sequences used for both cloning and sequencing, which are indicated in blue: the forward primer (5' CACCATGGGAGATACAACATCATCG) and the reverse primer (complementary sequence CCAGATCAAGATTATATTAAATTGAATTGTTAA 3') for cloning *exd3-1* gene, and an internal gene-specific primer (GAAACTGATGAGACGGTCAATG) for sequencing.

The sequencing data: 1-2355

5' CACC

1

ATGGGAGATACAACATCATCGGAAGATGTTCCGGAAAATAAGCAAAAATCACTGAAGTTTGAGATT
ATTGATGCAAGAATGAAGATTTTCAAAGATATAGTCAAAAGTAAATCAAGTGAATCAGTGAAGGAG
GAACAAATATATCAGAAGTCTCTGGAATTCTTTGATGAAGATCTCAAAAGTTC**T**GAAGAGTCTGTT
TCCAATGAAGAAATTAAAACGGGTTCGGAAAAAGAATTAGAACCATTGAATGTTTTTGATATAATT
CTGATTATGCTACAACAACTTCCGGAGCGAAAGAAACCCATTGGATCGCTTTTATCAAAATGGATT
CTGATGAATTTCATGAATTGGATGCAAGATAAGCAAAGCATTATGGAACAACAAATGACAGAATAT
TATCAAAAGAAGGCGGGACTTGCATGTGTTAAGGAGCCAAAAAATGAAGAATACTTACTACAAATC
TTCAAGATTTCAAAAGAATTTGTGATTGACTTGAGAAAATCGAAAGTTCAAGAATATCTTGAAAAT
CAAAAATTCAAAGAAGCAGCTGAAATTGTTATGAAACATGAAGTAGTTGATGATTATTCATTTGAA
CAAATTACCCTTCCACTTATCCTTTGTGATAAGGTTCAAATAGTTGATGAACTTCTTAAAATAAGT
AAGAAACTTCAAAAATCATACATCTCATTTCTTGATCAATTTGTGGCT**GAAACTGATGAGACGGTC**
AATGCATTTTTTGAACCGTATAAGGAAAAAGGAATGGTTACAATCAATTTGAGCCGATTTCATGGA
AAATCTCTAACTATTTTTATGCAAAAGTTTTTTAATGGACAAGTGAAACAATTCAAGTTTGATCTG
GAGGAGAGGAGAGATGCACCGAAATTTGTAGCAAATATGAAGAGAAAGGCATTGAAATATTTTGTT
GGCAAGCGATTTGAAGATCACGAAATGAATGATGAATTGTTTTGTGAGCACATGAAGAGTACATTA
CCTGAATGCACGGATAAAACGATAGTCCAGTTTTTGATTTTATTGTGGGACACTTGTATTTATGAA
AGAAGAATTGAAGCATTGTTCTGGGCAACTTATTCGAATATCGACCGTAATTCAAAATATATGCCA
CCCGATTTAAAAGAAGAACTTGAAAATCCAACTACCGAAATGAAAAACGGTTTGGAAAAGTTGCAG
GCATTGAGAACAACAAAAAATTGTCAGGAAGAAGACGAACAACTGTATGTGTTTGAGGAACAAAAG
AAATATCCTATTCGAATTGTGCAAAACGAGCAAGATTTGGAAATTCTGTTGTCTGAACTTGGAGAA
CTGGAAGAGGGGATGTATATTGGATATGATTCTGAGTTTAAACCATATCATTTGATAGATGTTAGC

ACATCAAGACTGGCAATCATTCAACTATTTTTCAAGGATAAAGCTTGGCTCATAAATTGTGTGGCA
ATTGACAATTTGGCATCTCGTGATGATGTATGGATTAGGCTCTATAAAGGTCTATTTGAATCAAAC
AAATTTAGTATAGTTGGATTTGATATTCGACAAGACATTGAAGCAATGTTCACTGTTCCTTCCATC
AACAAAAATTTCAAAATTGAAAATATTCAAAATGTAATCTGTGTGAAATCTCTGGCTGAAAATGTG
AATGCATTATCGATGGATATTTTGAATCTCAGCACGAAAACAAGTAAATTGTCAGTACTTGCTGAT
CATCTTGTTGGATTGAAAATGGATAAATCAGAACAATGTGGAAATTGGCAGTGTCGTCCATTGAGA
AGAAATCAGATAATCTACGCTGTAATGGATGCGGTCGCTGTTTTTGAAGTATTTCAAAAAATAGTG
GAAGTAGTTAGAAAACACGAATTGGATGCGGAAAAGTTGTTGGTCGAATCACATATGATTACTGTG
AAGAAAGAAAAAGTTAGGAGGGATTGTAAAAATATTAGTTTGATTCCGTGGAATGAGTTTTATCAG
ATTATCCACACCCATCGAAATCCAGAAAAACCGCTCCAAAAGCCATCTGAACTGAAAATAGTGGTG
GACACAATGGTTCTTGGATTGGGAAAAAATCTTCGATTACTCGGATTTGATGTCTACATTCCACGT
GATGTGACAGAGTTGAAAGAGTTTTTGAGGAAAATGGATAAAATGGAAGAATCCGAACAAAGATTA
GTTATCAGTGTTCCTTCGAGAAGTTATGAAATGTTGAAATCAGATAATCCTAATGCAAAGTTTGTA
TTGATTCCGAATATTTACGAAAAAGTACCCATTGATCTCGTTTGTTCATTTTTTGATTTTTTCAAC
ATTGATATTAGC**CCAGATCAAGATTATATTAAATTGAATTGTTAA** **3′**

2355
