# Supplementary material for: A new Caenorhabditis elegans apurinic/apyrimidinic (AP) endonuclease engaged in rescue from replication stress-induced arrest
Source: Genet Mol Biol. 2025 Oct 31;48(3):e20240216. doi: 10.1590/1678-4685-GMB-2024-0216 (PMC12582537; doi:10.1590/1678-4685-GMB-2024-0216)
Supplement: Figure S2 - [file 1415-4757-GMB-48-3-e20240216-s3.pdf]

Genetics and Molecular Biology

https://doi.org/10.1590/1678-4685-GMB-2024-0216

Supplementary Material to: A new *Caenorhabditis elegans* purinic/apyrimidinic (AP) endonuclease engaged in rescue from replication stress-induced arrest

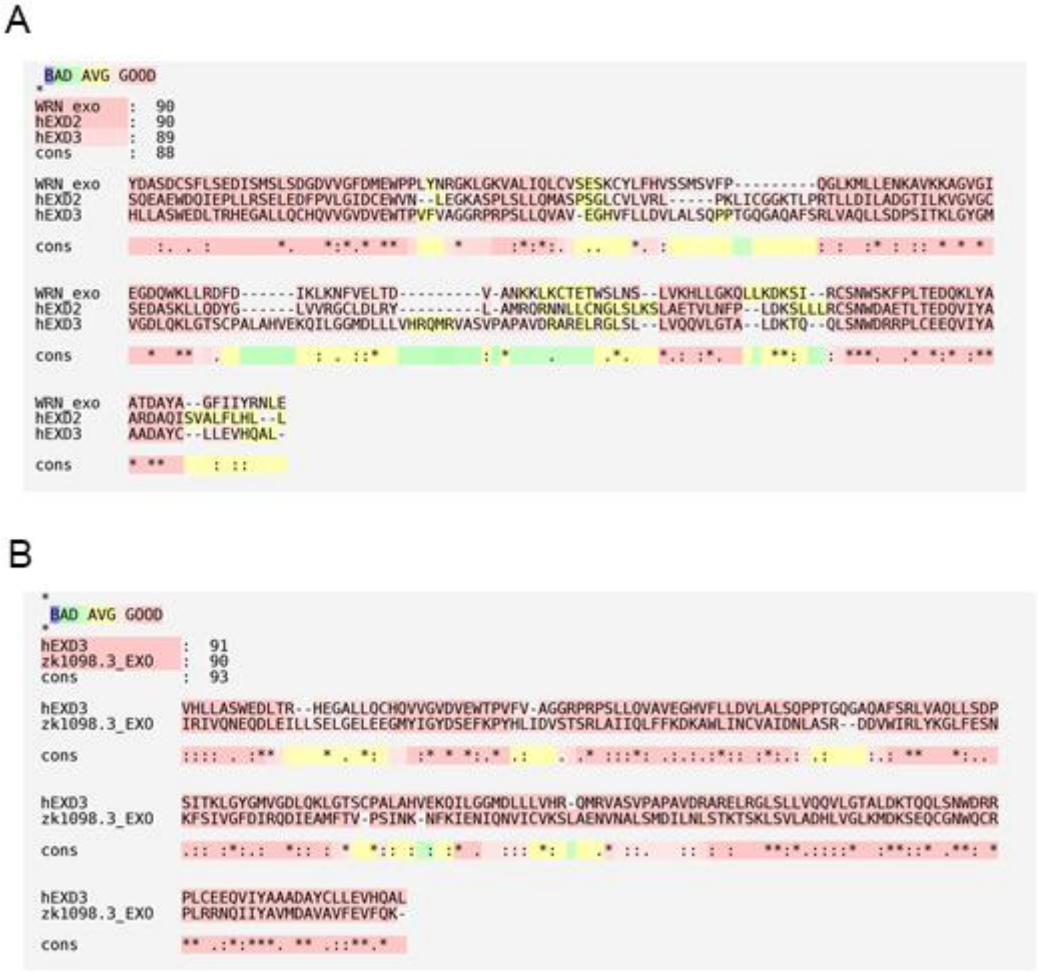

Figure S2 - Multiple sequence alignment of 3'-5' exonuclease domains in various proteins.

(A) Multiple sequence alignment of exodomains in WRN, human EXD3, and human EXD2. The WRN exo domain (amino acids 57-235) was aligned with corresponding exo domains in EXD3 (amino acids 373-568) and EXD2 (amino acids 82-258). Red portions have a high reliability and are expected to be more accurate that the rest. Blue and green portions are the less consistent. (B) A sequence alignment between ZK1098.3 (amino acids 382-619) and EXD3 (amino acids 373-568). Multiple sequence alignment was performed with Expresso, an alignment method through structural information (available at https://tcoffee.crg.eu/apps/tcoffee/do:expresso). Cons lines refer to the consensus or consistency of each aligned column across all sequences, indicating how well-conserved each column of the multiple sequence alignment is. Asterisk (*) indicates fully conserved column (all residues are identical). Colon (:) indicates strongly similar residues (same group, e.g., hydrophobic). T-Coffee-specific consistency scores scale goes from 0 (blue) to 9 (red). Each score (as an average score) from the consistency score across multiple positions reflects how well those residues align with the residues from the other sequences. A range of 90–100 indicates very likely correct alignment. A range of 70–89 indicates well-aligned region. Cons value is combined consistency score (for all comparisons).
