# Supplementary material for: A new Caenorhabditis elegans apurinic/apyrimidinic (AP) endonuclease engaged in rescue from replication stress-induced arrest
Source: Genet Mol Biol. 2025 Oct 31;48(3):e20240216. doi: 10.1590/1678-4685-GMB-2024-0216 (PMC12582537; doi:10.1590/1678-4685-GMB-2024-0216)
Supplement: Figure S3 - [file 1415-4757-GMB-48-3-e20240216-s4.pdf]

Genetics and Molecular Biology

https://doi.org/10.1590/1678-4685-GMB-2024-0216

Supplementary Material to: A new *Caenorhabditis elegans* purinic/apyrimidinic (AP) endonuclease engaged in rescue from replication stress-induced arrest

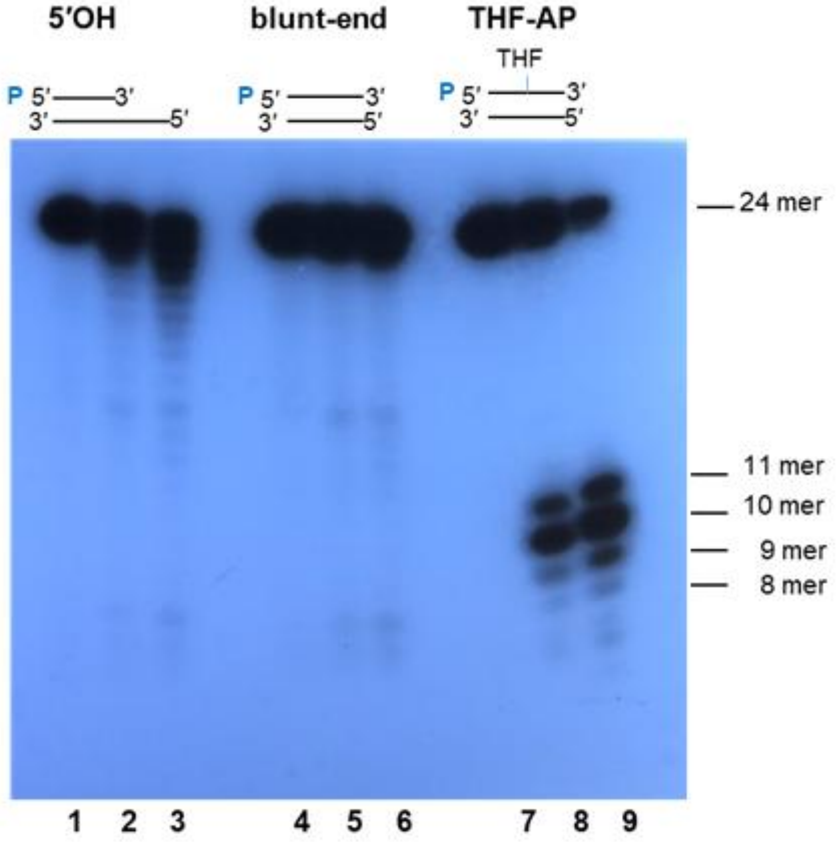

Figure S3 - Nuclease activities of EXD3-1 on various duplex DNA substrates.

Nuclease activity of EXD3-1 on three different duplex DNAs: a 24-bp duplex with 12 nt 5′-overhang (5′ OH) labeled with [γ-^{32}P] ATP at 5′ end of untailed strand (lanes 1-3), a 24-bp blunt-ended duplex labeled with [γ-^{32}P] ATP at 5′ end (lanes 4-6), a 24-bp duplex THF-AP substrate (lanes 7-9). Reaction was initiated with duplex DNA (100 fmol) and EXD3-1 for 30 min at 37°C for 30 min. Reaction products were separated by 8 M urea-17% PAGE (gel length 16 cm) and DNA fragments were visualized by x-ray film autoradiography. Lane 1; DNA substrate only, lane 2; EXD3-1 (300 fmol), lane 3; EXD3-1 (600 fmol), lane 4; DNA substrate only, lane 5; EXD3-1 (300 fmol), lane 6; EXD3-1 (600 fmol), lane 7; DNA substrate only, lane 8; EXD3-1 (300 fmol), lane 9; EXD3-1 (600 fmol).
