# Supplementary material for: A new Caenorhabditis elegans apurinic/apyrimidinic (AP) endonuclease engaged in rescue from replication stress-induced arrest
Source: Genet Mol Biol. 2025 Oct 31;48(3):e20240216. doi: 10.1590/1678-4685-GMB-2024-0216 (PMC12582537; doi:10.1590/1678-4685-GMB-2024-0216)
Supplement: Figure S4 - [file 1415-4757-GMB-48-3-e20240216-s5.pdf]

Genetics and Molecular Biology

https://doi.org/10.1590/1678-4685-GMB-2024-0216

Supplementary Material to: A new *Caenorhabditis elegans* purinic/apyrimidinic (AP) endonuclease engaged in rescue from replication stress-induced arrest

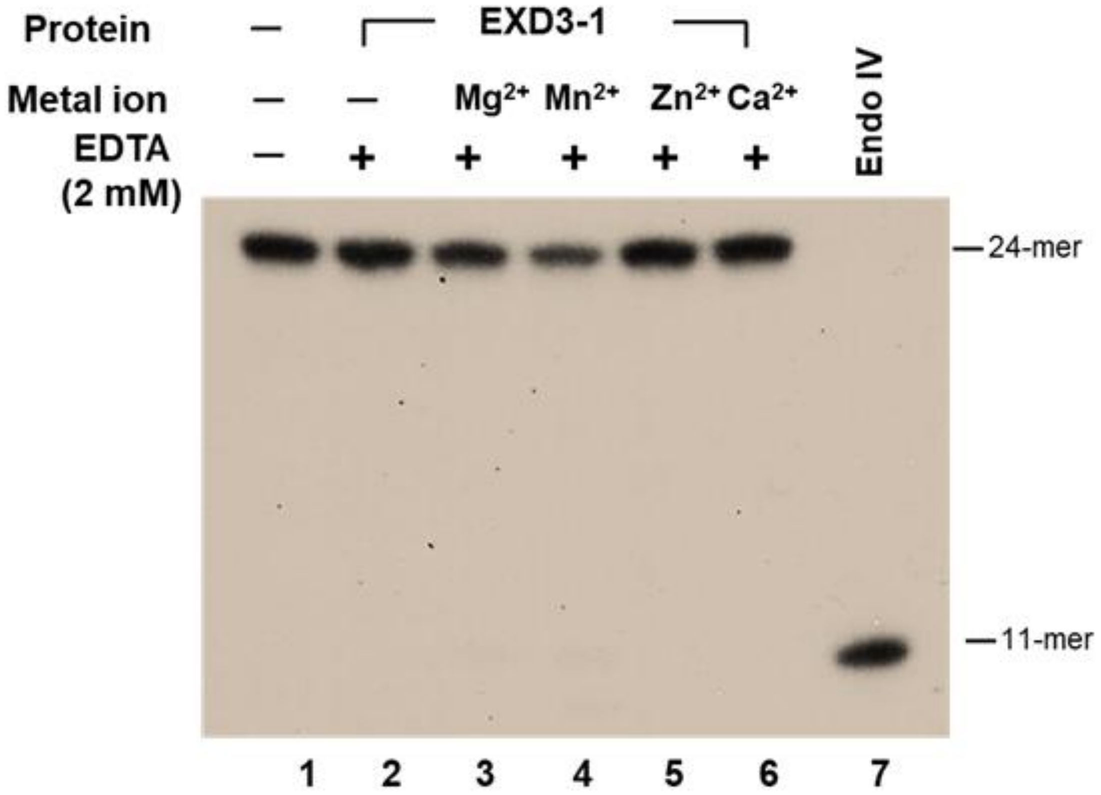

Figure S4 - Effects of EDTA (metal ion chelator) and divalent metal ions on incision of AP site by EXD3-1.
THF-AP substrate (100 fmol) was incubated with EXD3-1 (450 fmol) in the presence of EDTA (2 mM) and/or each of the indicated metal ions (4 mM). Reaction products were separated by 8 M urea-17% PAGE and DNA fragments were visualized by autoradiography. The length of the fragment is indicated on the right side of the autoradiography. Lane 1, only DNA; lane 2, EDTA and EXD3-1; lane 3, EDTA, $MgCl_2$, and EXD3-1; lane 4, EDTA, $MnCl_2$, and EXD3-1; lane 5, EDTA, $ZnCl_2$, and EXD3-1; lane 6, EDTA, $CaCl_2$, and EXD3-1; lane 7, *E. coli* Endo IV (0.1 U).
