# Supplementary material for: A new Caenorhabditis elegans apurinic/apyrimidinic (AP) endonuclease engaged in rescue from replication stress-induced arrest
Source: Genet Mol Biol. 2025 Oct 31;48(3):e20240216. doi: 10.1590/1678-4685-GMB-2024-0216 (PMC12582537; doi:10.1590/1678-4685-GMB-2024-0216)
Supplement: Figure S5 - [file 1415-4757-GMB-48-3-e20240216-s6.pdf]

Genetics and Molecular Biology

https://doi.org/10.1590/1678-4685-GMB-2024-0216

Supplementary Material to: A new *Caenorhabditis elegans* purinic/apyrimidinic (AP) endonuclease engaged in rescue from replication stress-induced arrest

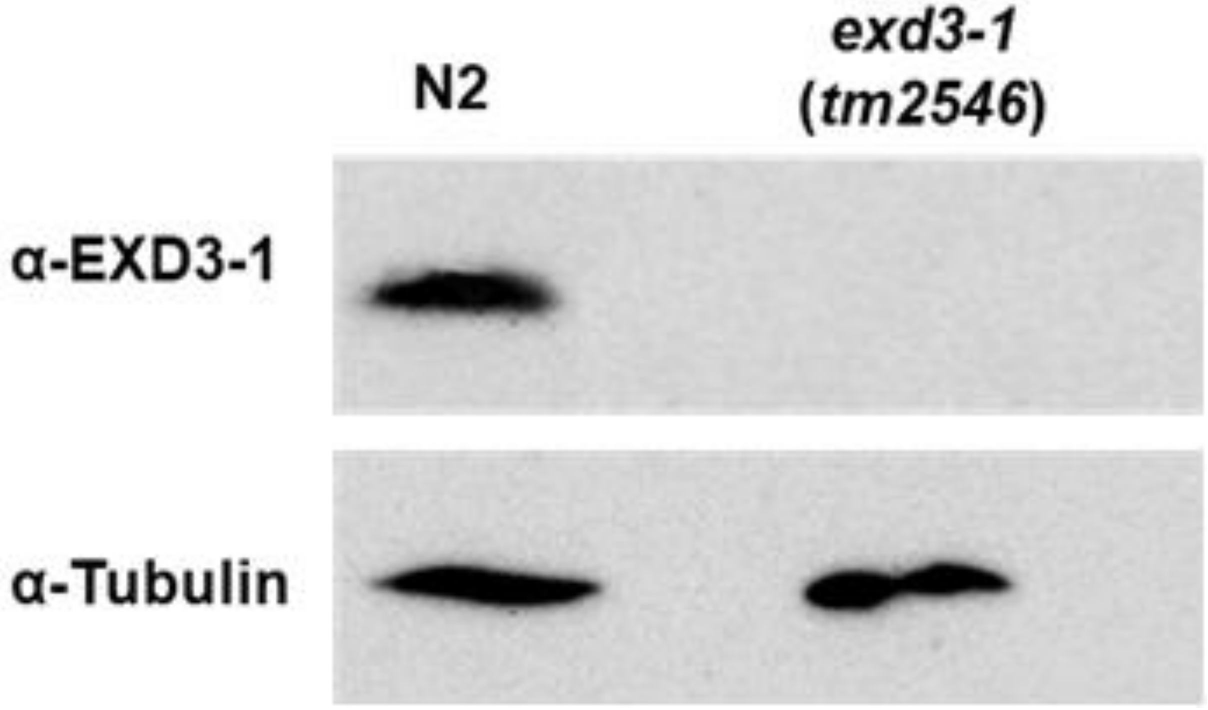

Figure S5 - Detecting EXD3-1 expression in worms by western blot analysis.

Worms were harvested from 10 plates with 1x PBST. Worms were washed three times with 1x PBST and were left in 50 ul of 1x PBST. Worm cell extraction buffer (2x; 123 mM Tris pH 8, 4% SDS, 10% glycerol. 10 mM DTT, 5 mM sodium Fluoride, 1 mM sodium orthovanadate) was added and then worms were sonicated in a water bath sonicator). Sonicated worms were heated at 95°C for 5 min to inactivate proteases. Protein concentrations were measured by Bradford assay. SDS-PAGE loading dye was added. Proteins were separated by 7.5% SDS-PAGE and analyzed by western-blotting for tubulin and EXD3-1. Worm extract (50 µg) was loaded.
