# Supplementary material for: A new Caenorhabditis elegans apurinic/apyrimidinic (AP) endonuclease engaged in rescue from replication stress-induced arrest
Source: Genet Mol Biol. 2025 Oct 31;48(3):e20240216. doi: 10.1590/1678-4685-GMB-2024-0216 (PMC12582537; doi:10.1590/1678-4685-GMB-2024-0216)
Supplement: Figure S6 - [file 1415-4757-GMB-48-3-e20240216-s7.pdf]

Genetics and Molecular Biology

https://doi.org/10.1590/1678-4685-GMB-2024-0216

Supplementary Material to: A new *Caenorhabditis elegans* purinic/apyrimidinic (AP) endonuclease engaged in rescue from replication stress-induced arrest

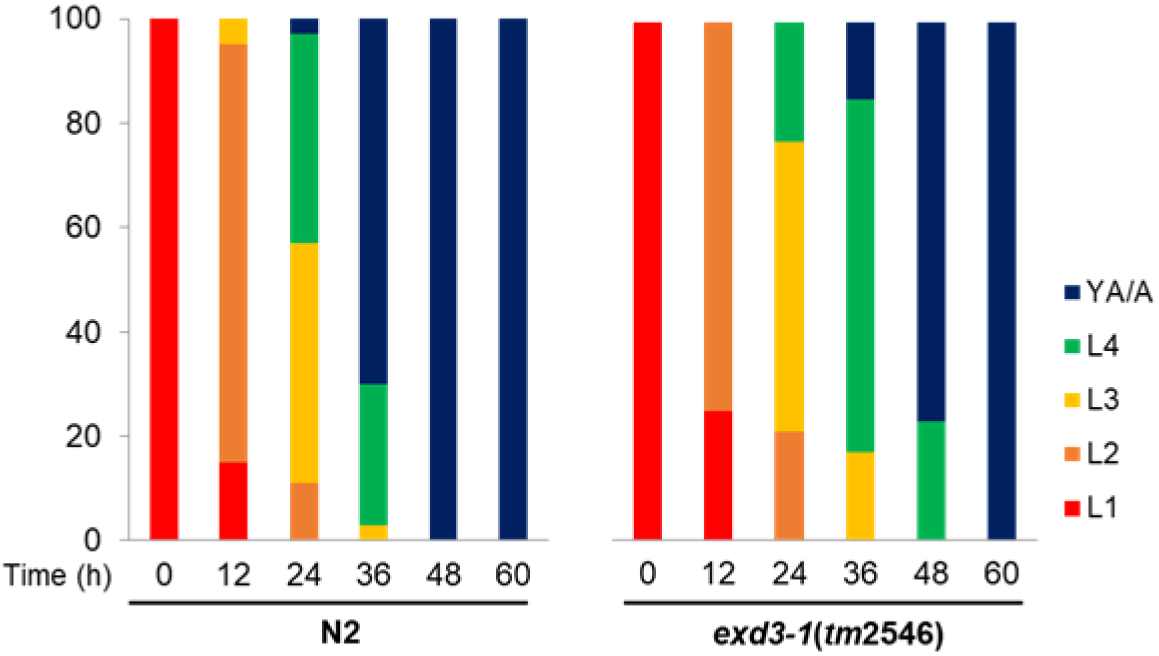

Figure S6 - Larval development of N2 and *exd3-1*(*tm2546*) worms.

Eggs (20–40) per plate were obtained by incubating two to three egg-laying (gravid) worms. Adult worms were discarded from plates and eggs used for experiments. Development from eggs to adult worms was monitored on NGM plates. The developmental stage of each worm was recorded by comparing the length of larva with that of N2 at multiple time points for 2–3 days. Each experiment was repeated 3 times.
