# Supplementary material for: A new Caenorhabditis elegans apurinic/apyrimidinic (AP) endonuclease engaged in rescue from replication stress-induced arrest
Source: Genet Mol Biol. 2025 Oct 31;48(3):e20240216. doi: 10.1590/1678-4685-GMB-2024-0216 (PMC12582537; doi:10.1590/1678-4685-GMB-2024-0216)
Supplement: Figure S7 - [file 1415-4757-GMB-48-3-e20240216-s8.pdf]

Genetics and Molecular Biology

https://doi.org/10.1590/1678-4685-GMB-2024-0216

Supplementary Material to: A new *Caenorhabditis elegans* purinic/apyrimidinic (AP) endonuclease engaged in rescue from replication stress-induced arrest

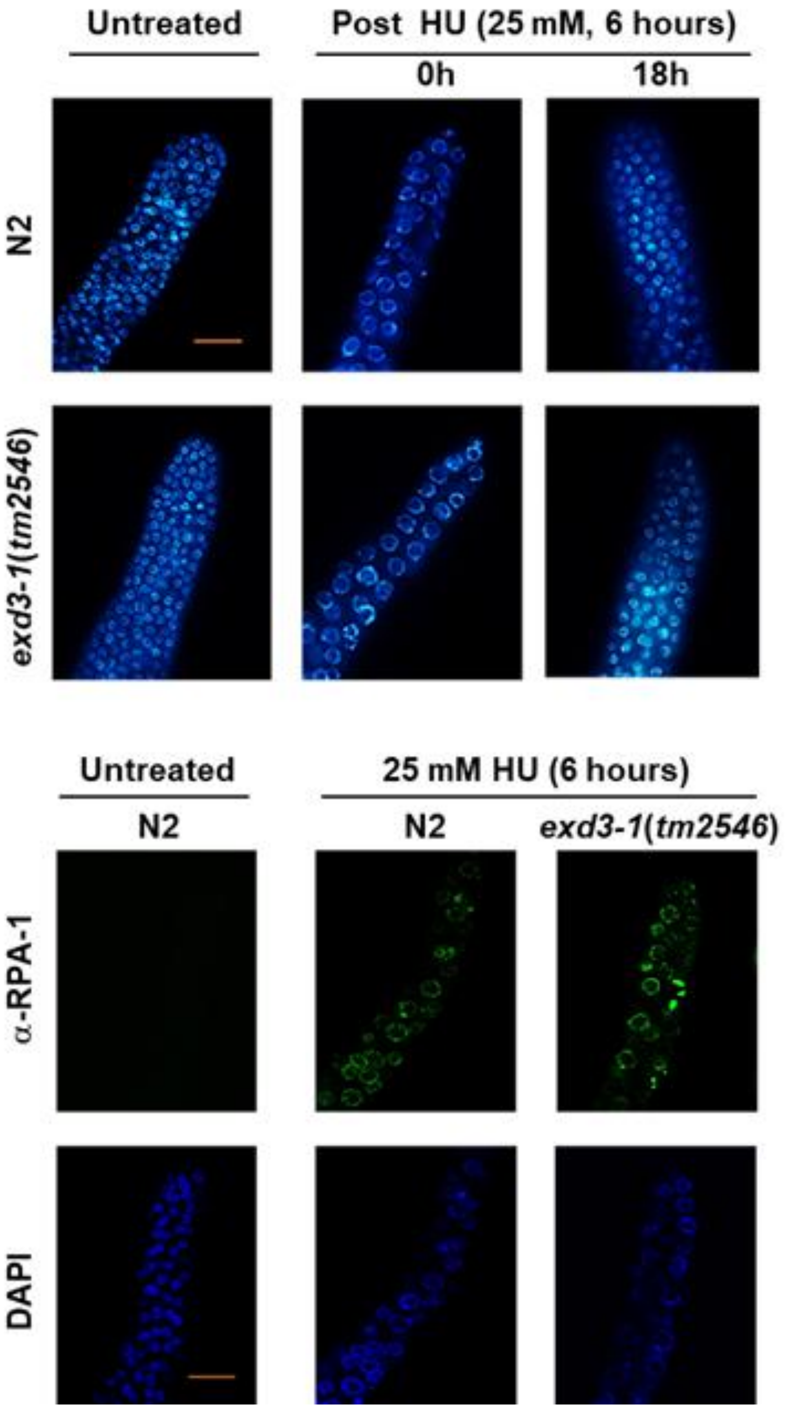

Figure S7 - RPA-1 foci were formed normally in *exd3-1*(*tm2546*) worms following 6-hour exposure to HU. Gonads of L4 stage worms were dissected and immunostained for RPA-1 6 hours after HU (25 mM) treatment and then stained with DAPI. Mitotic regions of *C. elegans* gonads were observed by fluorescence microscopy. Scale bars, 10 µm.
