# Supplementary material for: A new Caenorhabditis elegans apurinic/apyrimidinic (AP) endonuclease engaged in rescue from replication stress-induced arrest
Source: Genet Mol Biol. 2025 Oct 31;48(3):e20240216. doi: 10.1590/1678-4685-GMB-2024-0216 (PMC12582537; doi:10.1590/1678-4685-GMB-2024-0216)
Supplement: Figure S8 - [file 1415-4757-GMB-48-3-e20240216-s9.pdf]

Genetics and Molecular Biology

https://doi.org/10.1590/1678-4685-GMB-2024-0216

Supplementary Material to: A new *Caenorhabditis elegans* purinic/apyrimidinic (AP) endonuclease engaged in rescue from replication stress-induced arrest

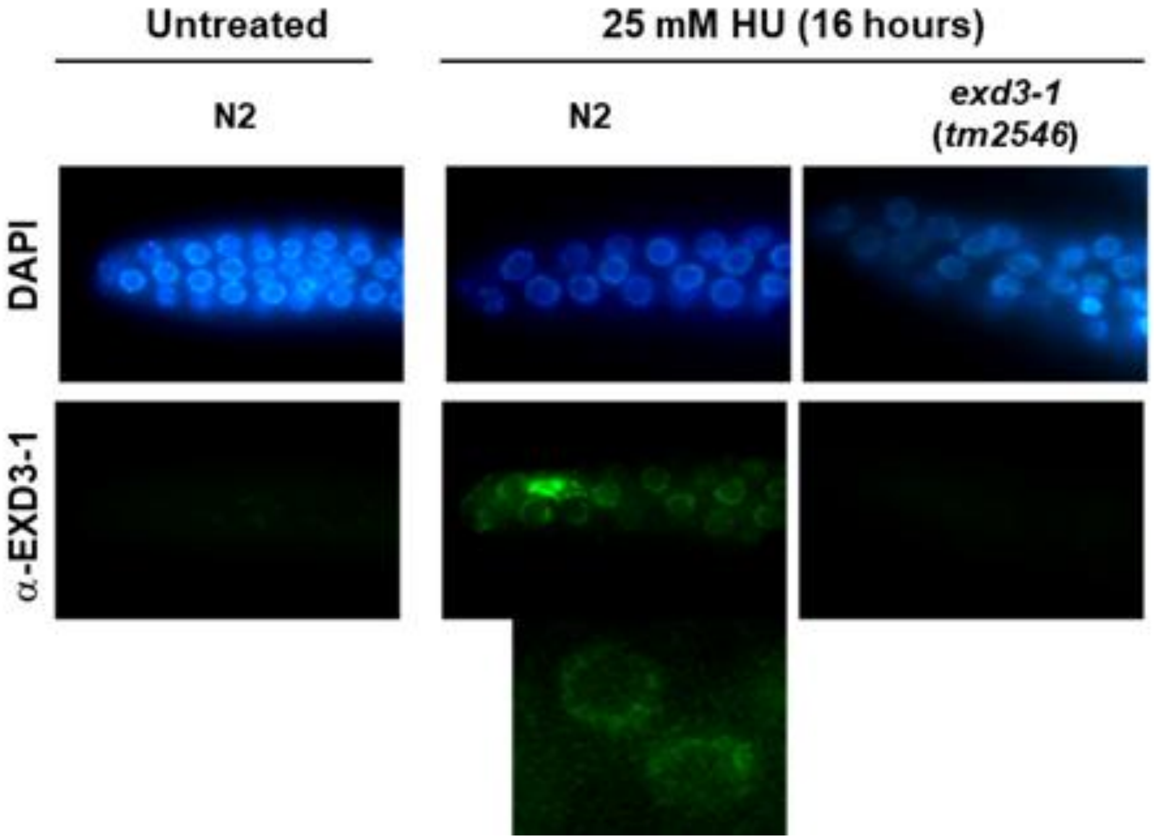

Figure S8 - Formation of EXD3-1 foci after HU treatment.

Representative images of fixed germ cells of indicated genotypes. L4 worms of N2 or *exd3-1*(*tm2546*) were dissected after 16-hour exposure to HU. The gonads were immunostained with anti-EXD3-1 antibody followed by staining with DAPI.
